# Uncovering the Anticancer Mechanism of Compound Sophorae Decoction against Ulcerative Colitis-Related Colorectal Cancer in Mice

**DOI:** 10.1155/2019/8128170

**Published:** 2019-10-20

**Authors:** Shuangjiao Deng, Qing Tang, Xueyun Duan, Heng Fan, Lijuan Zhang, Xiwen Zhu, Jianli Hu, Meng Xu, Qianyun Chen, Yujin Liu, Yalan Dong, Zhen Nan, Hui Wu

**Affiliations:** ^1^Department of Integrated Traditional Chinese and Western Medicine, Union Hospital, Tongji Medical College, Huazhong University of Science and Technology, Wuhan 430022, China; ^2^Department of Pharmacy, Hubei Provincial Hospital of Traditional Chinese Medicine, Wuhan 430061, China; ^3^Department of Pharmacy, Hubei Province Academy of Traditional Chinese Medicine, Wuhan 430074, China; ^4^Cancer Center, Union Hospital, Tongji Medical College, Huazhong University of Science and Technology, Wuhan 430022, China; ^5^Department of Geriatrics, Hubei Xinhua Hospital, Wuhan 430015, China

## Abstract

Compound sophorae decoction (CSD), a traditional Chinese medicine (TCM) formula, has been voluminously used in China to deal with ulcerative colitis and gained significant therapeutic effect. Tremendous explorations have unraveled a contributory role of inflammatory bowel disease (IBD) like ulcerative colitis (UC) and Crohn's disease (CD) at the onset of colorectal cancer, scilicet, and colitis-related cancer (CRC). In light of the anti-inflammatory properties of CSD in UC, we appraised its chemoprevention capacity and underlying mechanism in ulcerative colitis-related colorectal cancer (UCRCC), employing a model of azoxymethane (AOM) plus dextran sulfate sodium- (DSS-) induced colorectal cancer (CRC) in C57BL/6 mice. Rapturously, our results illuminated the ameliorative effect of CSD against UCRCC in mice portrayed by lesser polyps or adenomas, attenuated colonic xenograft tumor growth in company with the preferable well-being of mice in contrast to the Model Group. We examined significant downregulation of proinflammatory cytokines such as TNF-*α*, NF-*κ*B, IL-6, STAT3, and IL-17 after exposure to CSD, with the concomitant repression of inflammation-associated proteins, including COX-2 and iNOS. Independent of this, treatment with CSD declined the proportion of T helper 17 cells (Th17) and protein level of matrix metallopeptidase 9 (MMP-9). Moreover, transmission electron microscopy (TEM) detected observably suppressed mitophagy in mice administered with CSD and that was paralleled by the pro-apoptotic effect as indicated by upregulating caspase-3 together with caspase-9 and deregulating B-cell lymphoma 2 (Bcl-2). In closing, these findings suggest CSD executes the UCRCC-inhibitory activity through counteracting inflammatory responses and rescuing detuning of apoptosis as well as neutralizing overactive mitophagy, concurring to build up an oncosuppressive microenvironment.

## 1. Introduction

Ulcerative colitis (UC) is a long-lasting and relapsing inflammatory intestinal disturbance, whose etiology and pathogenesis is still elusive, with a growing body of compelling evidence defining its promoting role in the initiation of colorectal cancer [1–3]. It has been well-acknowledged that long-standing proinflammatory cytokines comprising tumor necrosis factor (TNF-*α*), interleukin- (IL-) 17, and NF-*κ*B/IL-6/STAT3 cascade in colon harbor a close relationship between UC and colon cancer and simultaneously behave as pivotal mediators in the onset and deterioration of UC and colorectal cancer [4–9]. Accordingly, these data underscore a crucial role of proinflammatory responses in the context of UCRCC and confer the urgency to address the related concrete mechanism.

Mitochondria are cardinal double membrane-bound organelles and cellular stress sensors involved in multifaceted cellular activities containing energy production, senescence, apoptosis, oxidative stress regulation, and metabolism in addition to signaling. Hence, these cellular properties of mitochondria denote a highly-associated link connecting cellular dysfunction in the context of cancer or noncancer and abnormalities in mitochondrial status along with activity and meanwhile, authenticate the magnitude of the timely elimination of damaged and aged mitochondria which is called mitophagy for maintaining the cellular integrity [10–17]. To our knowledge, cancer is a disturbance in the homeostatic balance between cell growth and cell death, which is featured by metabolic reprogramming, uncontrolled cellular proliferation, and enhanced resistance to apoptosis of tumor cell. Massive reports [10–12, 16, 17] have identified that mitophagy is activated under conditions of stimuli such as nutrient depletion, hypoxia, and activated oncogenes, imparting considerable flexibility for tumor cell growth and survival. In addition, mitochondria-targeted drugs and targeting apoptosis pathways open up the opportunity for the development of novel therapeutic strategies for cancer abrogation [10, 11]. Therefore, it is anticipated that a broad understanding of mitophagy and apoptosis in UCRCC may shed light on investigating the tumor-promoting mechanisms to the next level.

Compound sophorae decoction (CSD) is a classical traditional Chinese medicine (TCM) preparation developed from qingre zaoshi liangxue fang (QRZSLXF) [18] has been widely applied in China to medicate UC patients and is clinically efficient [19, 20]. Kushen, videlicet, *Sophora flavescens* Ait., is the sovereign drug of CSD and is used extensively to treat fibrosis, asthma, inflammatory disorders, ulcers, and solid tumor [19–26]. However, definitive mechanisms that demonstrate the role of CSD in UCRCC are still obscure. Thereby, further studies analyzing the contribution of CSD to the amelioration of UCRCC and identifying its active ingredients via mass spectrometry (MS) appear warranted.

**Figure 1:**
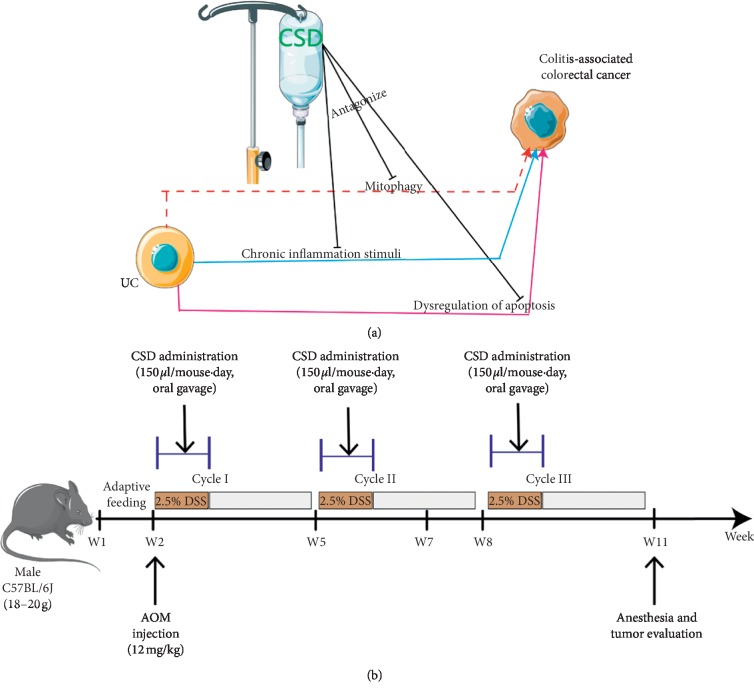
A schematic diagram of the workflow of CSD in UCRCC (a) and experimental protocol for UCRCC model and validation for investigation of the mechanisms of CSD (b).

Taken together, we hypothesize that inflammatory responses along with uncontrollable homeostasis between mitophagy and cellular apoptosis synergistically facilitate the formation of ambience in favor of UCRCC, and CSD overturns the tumourigenesis effect (Figure 1(a)). This study may provide novel insights into the carcinogenesis of UCRCC and open a promising therapeutic approach to UCRCC.

## 2. Materials and Methods

### 2.1. Animals and Mouse Model of UCRCC

Male C57BL/6J mice (6–8 weeks old) were lodged under specific pathogen-free (SPF) conditions with free access to autoclaved food and water in the experimental animal center of Huazhong University of Science and Technology (HUST, Wuhan, China). They were stochastically grouped into Model Group (AOM/DSS), CSD Group (AOM/DSS + CSD), and Normal Group. UCRCC model was conducted based on a typical protocol [27, 28], that is, the administration of a single intraperitoneal injection of AOM (12 mg/kg, Sigma) in conjunction with three rounds of 2.5% DSS (36–50 kDa; MP Biochemicals) application (Figure 1(b)). All animal care and experimental processes were performed in accordance with guidelines of the Animal Research Institute Committee of HUST and National Institutes of Health guidelines and regulations.

### 2.2. Composition and Preparation of CSD

CSD is a Chinese herbal mixture composed of *Sophora flavescens* Ait. (15 gram), Radix Sanguisorbae (15 gram), Indigo Naturalis (3 gram), *Bletilla striata* (Thund.) Reichb. f. (10 gram), *Panax notoginseng* (Burk.) F. H. Chen (3 gram), and *Glycyrrhiza uralensis* Fisch. (10 gram). All the raw herbal medicines were purchased from Hubei Provincial Hospital of Traditional Chinese Medicine (Wuhan, China) and then mixed according to the weight ratio before soaking for 1 h. Eventually, the mixture was condensed into a concentration of 1.076 g/ml as CSD and stored at 4°C after undergoing initial hard boil and being simmered for 1 h and incurring succedent filter. 150 *μ*l CSD was administrated by gavage daily, synchronizing the procedure of DSS induction.

### 2.3. Behavioral and Physiological Assessment

Body weight, stool consistency, and hemafecia ratio in addition to intake of food and water were recorded daily throughout the whole span of the experiment. After figuring out weight and length of colons, the number and diameter of tumors were calculated.

### 2.4. Western Blot Analysis

Proteins of each colon were extracted in RIPA buffer supplemented with phosphatase and protease inhibitors. 40 *μ*g proteins were utilized for the investigation of inflammatory responses in the colon as described previously [29]. Antibodies recognizing the proteins were as follows: anti-IL-6 (1 : 500, Bioss, Beijing, China), anti- TNF-*α* (1 : 1000, Abcam, Cambridge, UK), anti-NF-*κ*B (1 : 2000, Cell Signaling Technology, USA), and anti-IL-17 (1 : 1000, Abcam, Cambridge, UK).

### 2.5. Histological Evaluation and Immunohistochemistry

Fresh colon sections were embedded in paraffin after being fixed in 4% paraformaldehyde and then were cut into 4 *μ*m slides that would be stained with haematoxylin-eosin (H&E) hereafter. An expert pathologist carried out histopathological examinations blindly. For immunohistochemical assessment, the paraffin-embedded colonic slides were subjected to immunohistochemical staining and incubated with primary antibodies for cyclooxygenase-2 (COX-2; 1 : 100, Cell Signaling Technology, USA), inducible nitric oxide synthase (iNOS; 1 : 100, Boster, Wuhan, China), matrix metallopeptidase 9 (MMP-9; 1 : 100, Ruiying Biological, Suzhou, China), TNF-*α* (1 : 50, Santa, Dallas, TX, USA), B-cell lymphoma 2 (Bcl-21 : 100, Boster, Wuhan, China), caspase-3 (1 : 100; PTG, Wuhan, China), and caspase-9 (1 : 100; Boster, Wuhan, China) complying with the manufactures' protocols.

### 2.6. Transmission Electron Microscopy Observation

For the transmission electron microscopic (TEM) analysis of mitochondria, pretreated colon tissues underwent a series of procedures reported as previously [10] and the stained ultrathin colonic sections (60–80 nm) were detected using a Hitachi-HT7700 electron microscope (Tokyo, Japan).

### 2.7. Flow Cytometry

After being stimulated with phorbol myristate acetate (PMA; Abcam, Cambridge, UK), ionomycin, and GolgiPlug protein transport inhibition (BD Biosciences, San Diego, USA) in a humidified 37°C and 5% CO_2_ incubator for 7 h, single-cell suspension of splenocytes and mesenteric lymph nodes (MLNs) were stained with FITC-labeled antimouse CD4 and PE-labeled antimouse IL-17A antibodies (BD Biosciences, MD, USA). Isotype antibody was adopted as the negative control. Thenceforward, the stained cells were washed and analyzed by using a FACSCalibur flow cytometer (BD Biosciences, San Diego, CA).

### 2.8. High-Resolution Metabolomics

100 *μ*l CSD liquid samples underwent methanol extraction by adding 900 *μ*l methanol or pure water extraction by adding 900 *μ*l pure water, following the procedures listed as follows: vortexing for 1 min; centrifuging for 10 min, 12000 r/min, 4°C; filtering the supernatant through a 2 *μ*m filter; and analyzing the filtrate on the machine. Untargeted metabolic profiling of CSD was performed employing high-resolution mass spectrometry (HRMS; Q-Exactive High-Resolution Mass Spectrometer, Thermo Fisher Scientific). Analyte separation was accomplished with the aid of liquid chromatography (UltiMate 3000 RS, Thermo Fisher Scientific) fitted with chromatographic column (Thermo Hypersil GOLD 100 × 2.1 mm, 1.9 *μ*m) manoeuvred at 0.3 mL/min with aqueous phase (0.1% aqueous formic acid) and organic phase (0.1% formic acid acetonitrile). The operating gradient came as follows: 0–2 min (aqueous phase: 95% ⟶ 80%, organic phase: 5% ⟶ 20%); 2–6 min (aqueous phase: 80% ⟶ 25%, organic phase: 20% ⟶ 75%); 6–8.5 min (aqueous phase: 25% ⟶ 5%, organic phase: 75% ⟶ 95%); 8.5–12.5 min (aqueous phase: 5%, organic phase: 95%); 12.5–13 min (aqueous phase: 5% ⟶ 95%, organic phase: 95% ⟶ 5%); and 13–16 min (aqueous phase: 95%, organic phase: 5%).

The electrospray ionization source was performed in positive ion mode with a spray voltage of 3.8 kV, capillary temperature of 300°C, sheath gas (nitrogen, purity ≥99.999%) flow of 40 arbitrary units (Arb), and auxiliary gas (nitrogen, purity ≥99.999%) temperature of 350°C. The resolution was set to 70000 (full mass), 17500 (dd-MS2), and the scan range was 70–1000 *m*/*z*. Data acquisition time was 16 min.

Mass spectral features represented by accurate mass *m*/*z*, retention time, and intensity were detected by high-resolution FTMS and sorted using CD2.1 software (Thermo Fisher) and then identified, aligned, and quantified according to databases such as Mzcloud, MzVault, and ChemSpider with the value of mzCloud Best Match ≥80%.

### 2.9. Statistical Analysis

All experimental data obtained from this study were presented as mean ± standard deviation (SD). Statistical significance between the data from different groups was calculated by one-way analysis of variance (ANOVA) or Student's *t*-test using SPSS software (version 19.0). A *p* value <0.05 was deemed as statistically significant.

## 3. Results

### 3.1. CSD Upgrades Clinical Symptoms in Mice Treated with AOM/DSS and Allays AOM/DSS-Induced Malignancy

**Figure 2:**
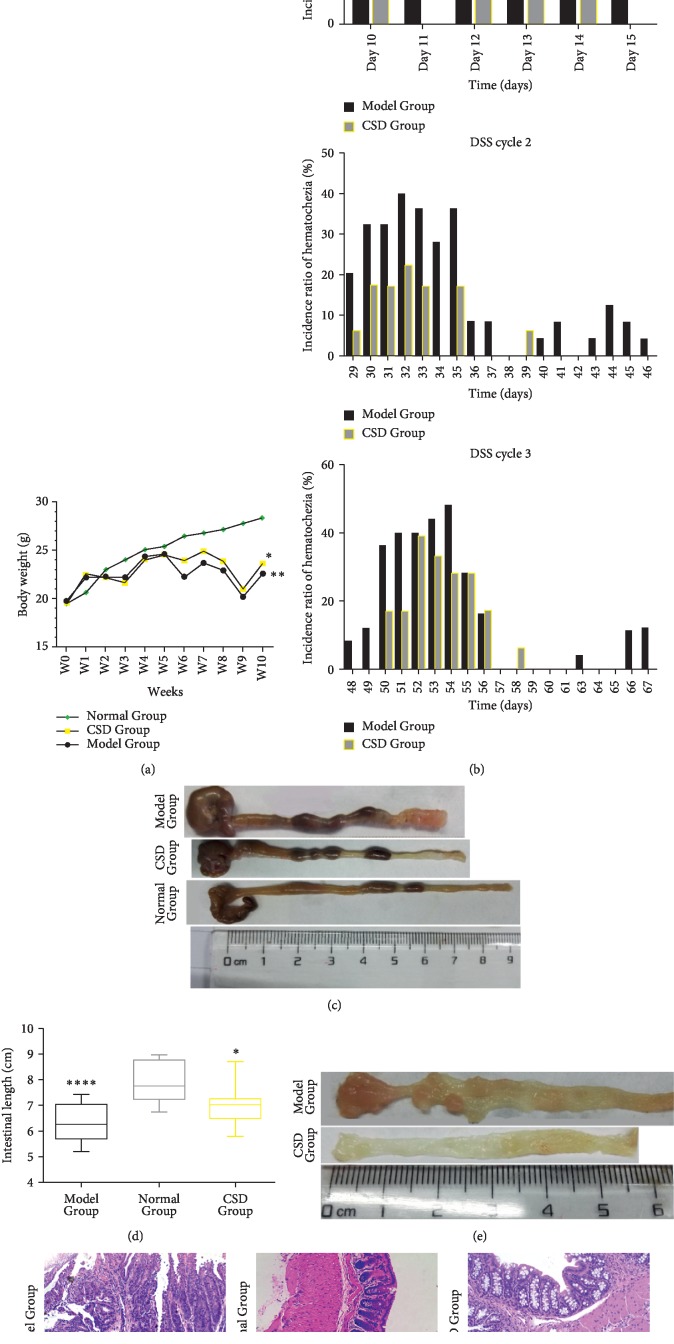
CSD overcomes AOM/DSS-induced malignancy of colon. (a) Body weight per mouse measured per day during all the experiment. ^*∗*^*p* < 0.05, ^*∗∗*^*p* < 0.01, significantly different from the Normal Group. (b) Effect of CSD on the incidence ratio of hematochezia. ^*∗*^*p* < 0.05 vs. Model Group. (c) Colonic length of mice from the three groups. (d) Comparison of colon length among the three groups, ^*∗*^*p* < 0.05, ^*∗∗*^*p* < 0.01 vs. Normal Group. (e) Effect of CSD on multiplicity of polyp/adenoma on colons. (f) Colon sections stained with H&E (×200) from each group.

As shown in Figure 2(a), significant body weight loss during the experimental period in mice from Model Group compared with Normal Group was alleviated by CSD administration, particularly after the third DSS cycle. In agreement with this, CSD treatment posed a decrement in the incidence ratio of hematochezia accompanied by postponed occurrence of diarrhea and blood in feces, as evaluated (Figure 2(b)). The shortening of colons, signifying the aggravation of colonic damage, was observed in mice exposed to AOM/DSS in comparison with mice in the CSD Group (*p* < 0.05) (Figures 2(c) and 2(d)). Furthermore, our data manifested higher polyp/adenoma multiplicity escorted by higher grade of epithelial dysplasia in the Model Group (Figures 2(e) and 2(f)).

### 3.2. CSD Moderates the Malignant Inflammatory Features in AOM/DSS-Induced UCRCC

**Figure 3:**
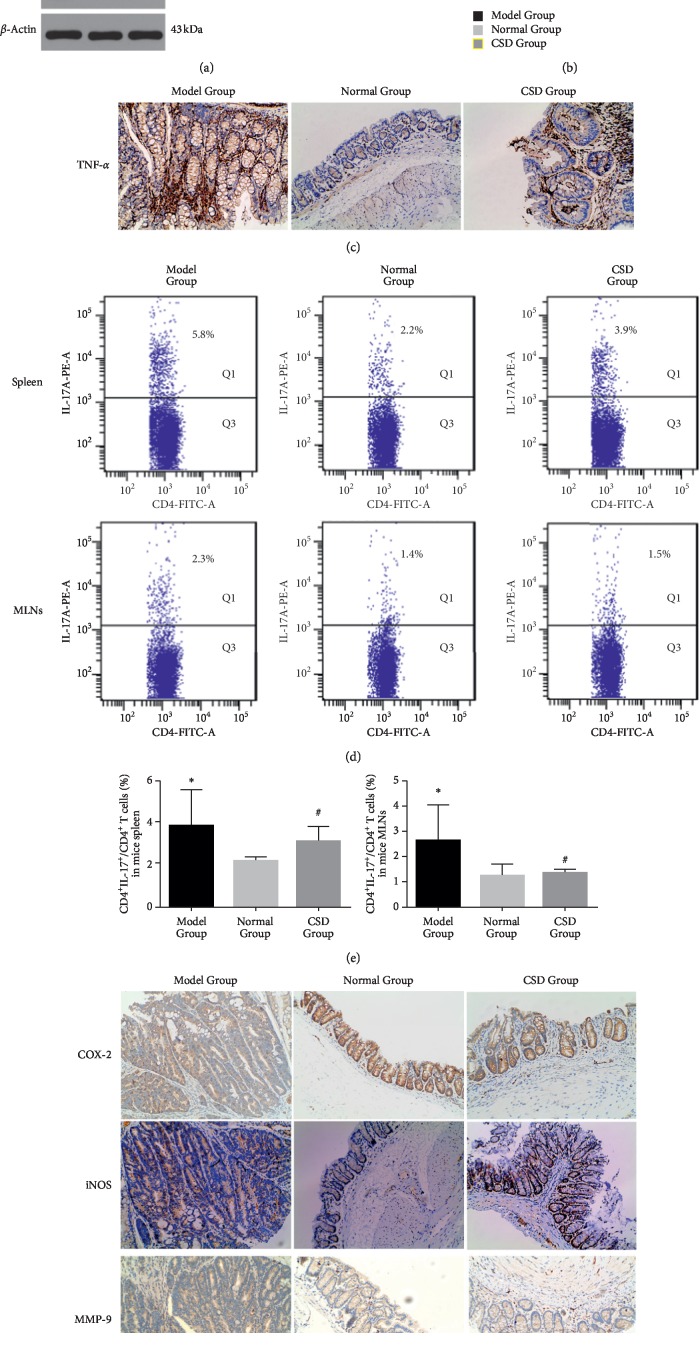
AOM/DSS-induced inflammatory response is impeded by CSD. (a) Western blot analysis of classical inflammatory proteins expression profiles in colon. (b) Representative histogram of densitometry analysis of the data derived from western blot, ^*∗∗*^*p* < 0.01, significantly different from the Model Group. (c) Immunohistochemical staining analysis of TNF-*α* expression in colons. (d) Representative flow cytometry dot plot of the percentages of CD4^+^IL-17^+^Th17 cells in CD4+ cells in the spleen and MLNs of each group. (e) Trends of Th17 cells in mice, ^*∗*^*p* < 0.05, ^#^*p* > 0.05 vs. Model Group. (f) Effect of CSD on COX-2, iNOS, and MMP-9 expression in colon tissues in C57BL/6 mice.

Incremental expression of proinflammatory cytokines such as NF-*κ*B and TNF-*α* and overactivation of IL-6/STAT3 passage have been well determined to exercise definitive implication in the pathogenesis of UC and colon cancer. In line with this, mice received AOM/DSS exhibited a rise in intestinal production of these parameters and IL-17 concentration in comparison with Normal Group, whereas intake of CSD significantly reversed the reaction expectably as illustrated by western blot and immunohistochemistry (Figures 3(a)–3(c)). Given the traditional role of Th17 cells in binding UC and UCRCC together, we explored the disparities in the proportion of Th17 cells isolated from spleens and MLNs of mice via flow cytometry (Figure 3(d)). Not surprisingly, the analysis revealed that Th17 cells may prompt the incipience of UCRCC and can be partially overthrown by CSD (Figure 3(e)). Regarding intestinal iNOS and COX-2 expression profiles, they were incremental in colon tissues from mice treated with AOM/DSS compared with the untreated mice, being diminished by treatment with CSD (Figure 3(f)).

### 3.3. CSD Modulates Ultrastructural Changes and Apoptosis in AOM/DSS-Induced Mice

**Figure 4:**
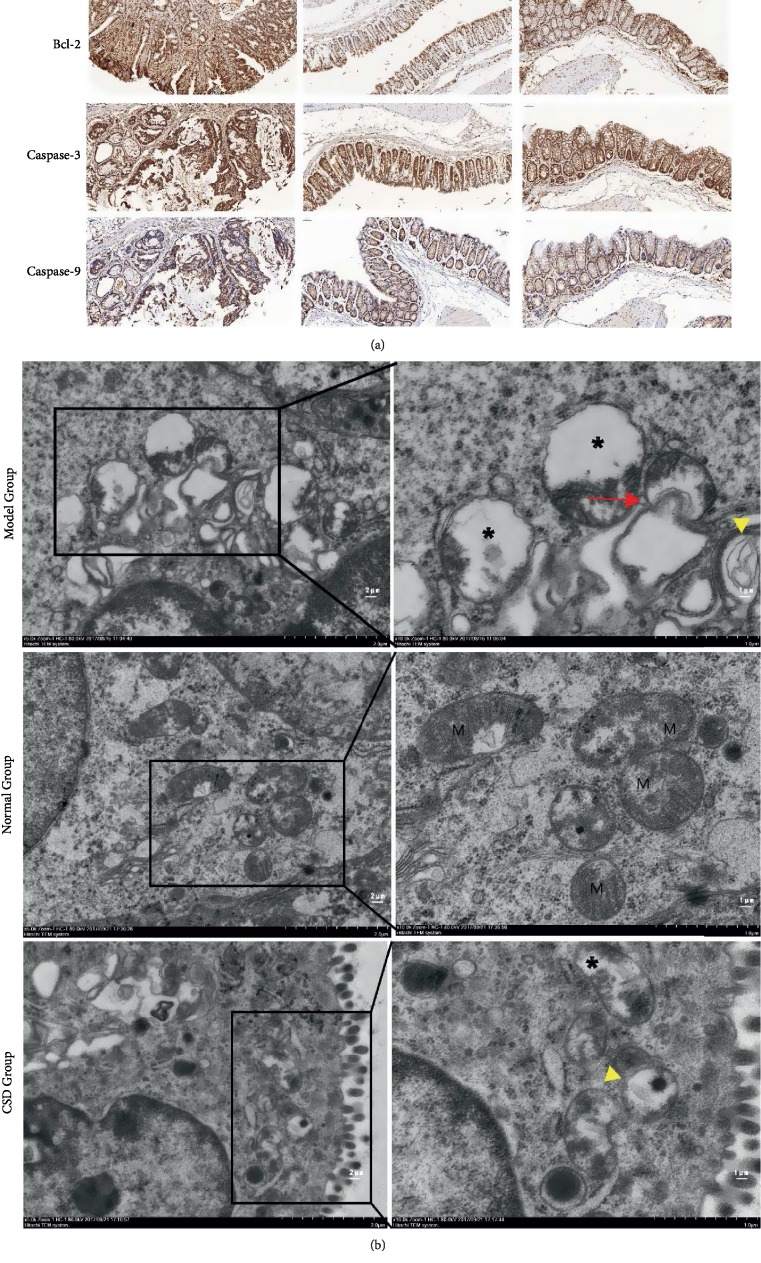
(a) CSD attunes apoptosis-related proteins expression language illustrated by immunohistochemistry. (b) Electron micrographs of mitochondrial morphology colonic tissues in mice. Arrowheads indicate the disrupted mitochondria undergoing mitochondrial fission, absence of cristae or electron-condensation; Arrows symbolize lysosomes engulfing damaged mitochondria. M means mitochondrion.

Apoptosis and mitophagy are two representative procedures that act in synergy to regulate cell survival and death in numerous types of cancer. To gain further insight into the pattern of CSD on apoptosis and mitophagy, we determined the changes in the levels of apoptosis regulatory proteins and mitochondrial morphology in the colons with the aid of immunohistochemical staining and TEM, respectively. The data came out with significant up-regulation of mitochondrial cleaved-caspase-3, caspase-9 and down-regulation of Bcl-2 in colons after implementing CSD therapy, evincing the apoptosis-encouraging efficacy of CSD (Figure 4(a)). We extended our attempts to probe mitochondrial structure from TEM images (Figure 4(b)), as demonstrated by the phenomenon that there was a pronounced increase in vacuolization (black asterisk panels), massive mitochondrial fission and loss of cristae as well as highly electron-condensation (arrowheads), and even lysosomes engulfing damaged mitochondria (arrows) in Model Group. To the contrary, the conspicuous mitochondrial morphological alternations brought on by AOM/DSS were perceptibly absent following CSD administration (Figure 4(b)), establishing mitophagy as an etiological factor in UCRCC tumorigenesis. The expression profile of MMP-9, whose well-appreciated pathologies is the relationship to cancer owing to its role in extracellular matrix remodeling and angiogenesis, was depicted to experience a slight diminution inflicted by CSD (Figure 3(f)).

### 3.4. Identification of Chemical Ingredients in CSD by Mass Spectrographic Analysis

**Figure 5:**
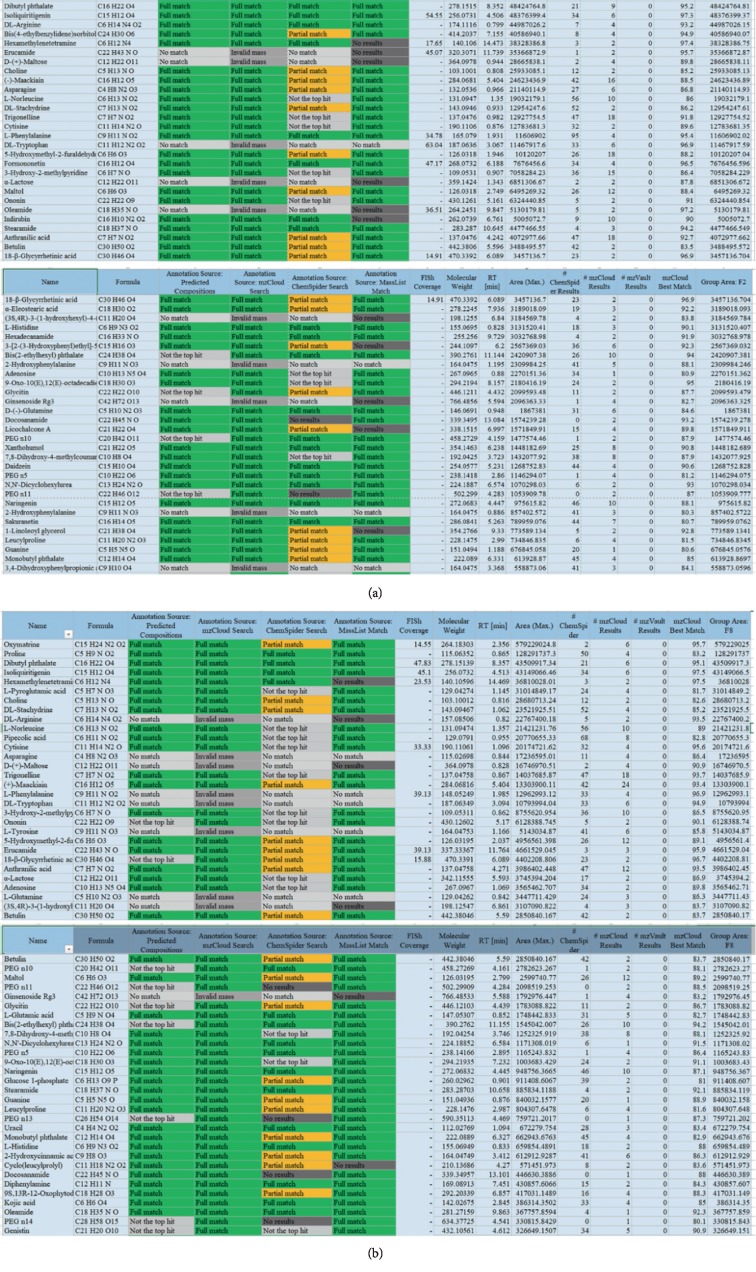
Detection of CSD by high-resolution FTMS analysis. Illustration of part of a summary of the various abundant constituents detected and identified in CSD by channel of (a) methanol extraction and (b) pure water extraction.

**Figure 6:**
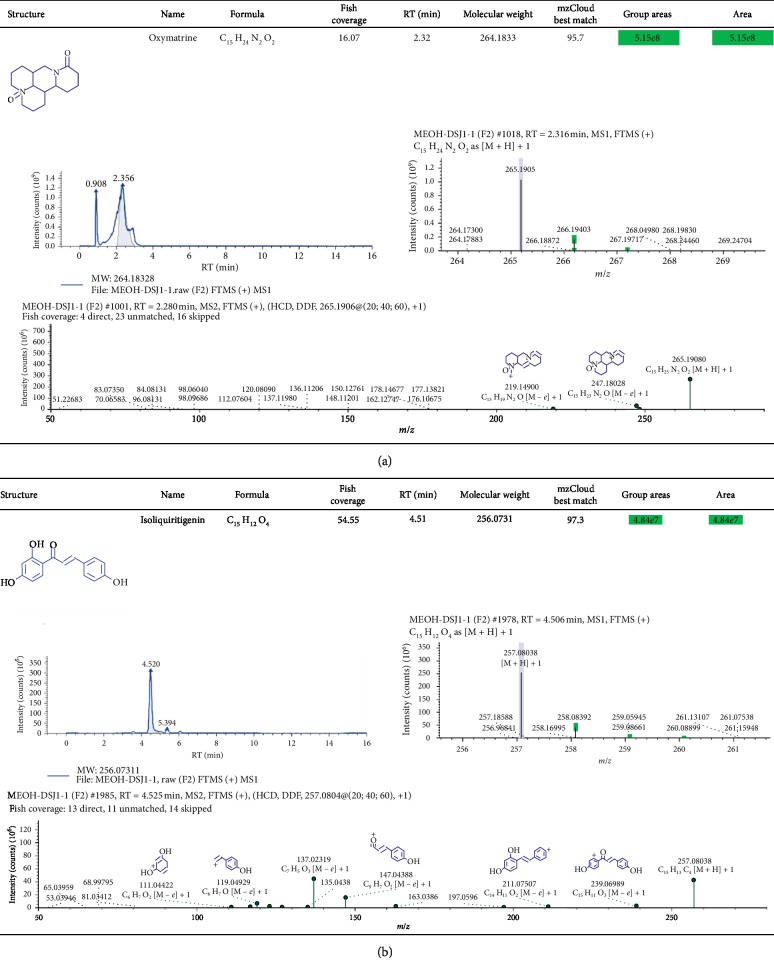
Positive-ion mode FTMS spectrum of the partial active ingredients from CSD extraction via methanol extraction. The active ingredients listed were oxymatrine (a) and isoliquiritigenin (b).

**Figure 7:**
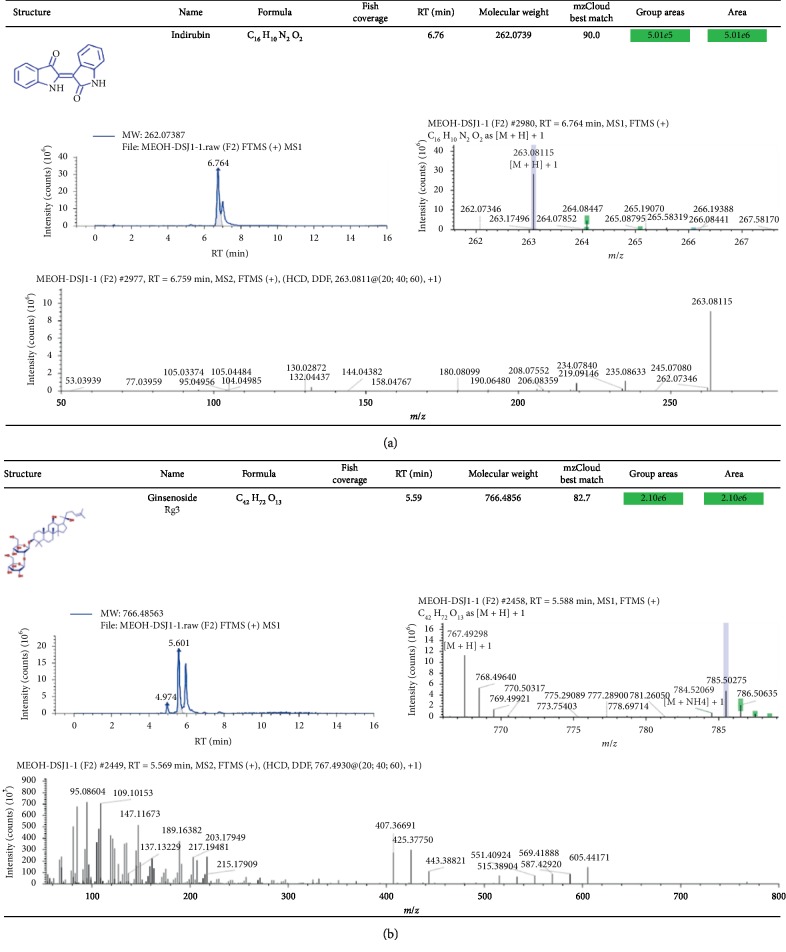
Positive-ion mode FTMS spectrum of the partial active ingredients from CSD extraction via methanol extraction. The active ingredients listed were indirubin (a) and ginsenoside Rg3 (b).

**Figure 8:**
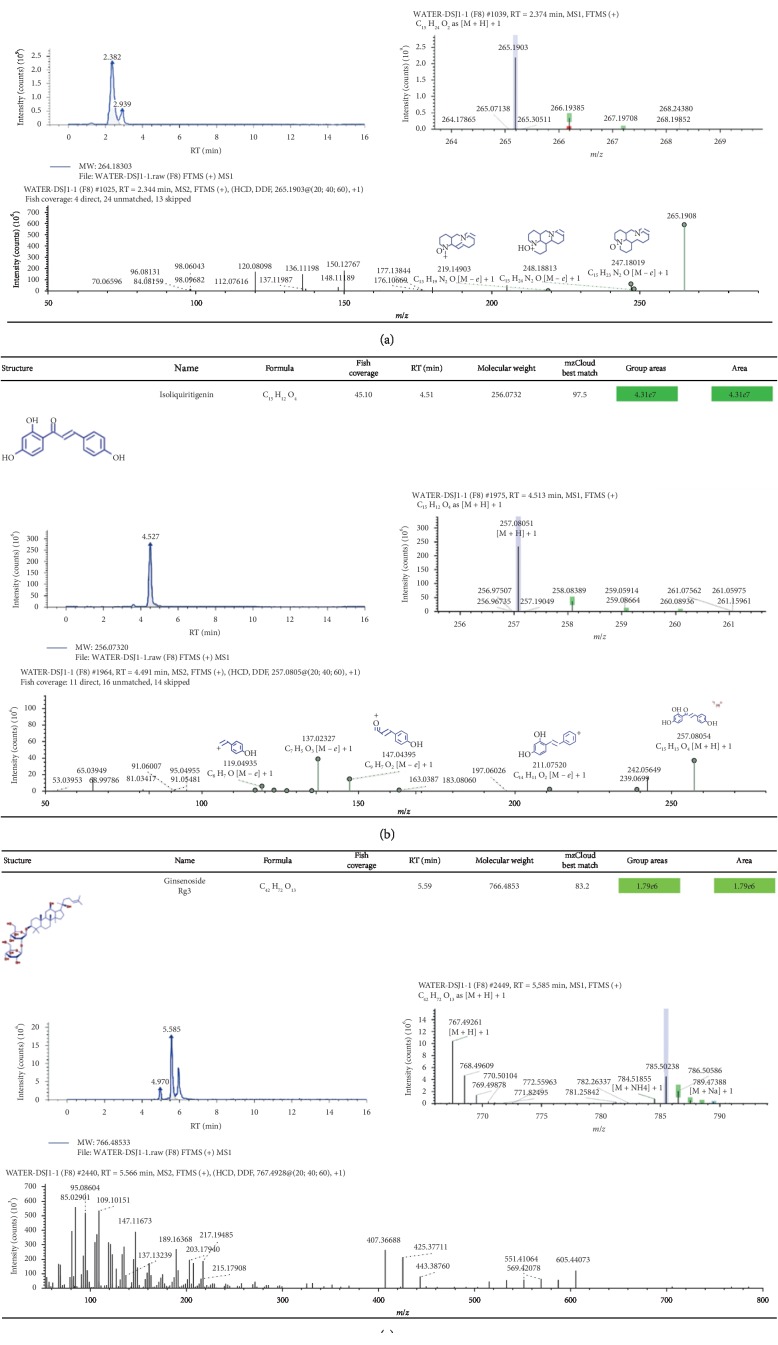
Positive-ion mode FTMS spectrum of the partial active ingredients from CSD extraction via pure water extraction. The active ingredients listed were oxymatrine (a), isoliquiritigenin (b), and ginsenoside Rg3 (c).

We attempted to identify the active constituents in CSD using high-resolution mass spectrometry (HRMS) and part of a summary of the various abundant constituents detected and identified by the channel of methanol extraction (Figure 5(a)) and pure water extraction (Figure 5(b)) was given, respectively. HRMS confirmed oxymatrine as the most abundant ingredient in the CSD liquid (Figures 5(a) and 5(b)). The active compounds from CSD extraction through methanol extraction were as follows: oxymatrine, isoliquiritigenin, (−)-maackiain, DL-stachydrine, cytisine, indirubin, 18-*β*-glycyrrhetinic acid, ginsenoside Rg3, licochalcone A, xanthohumol, 7,8-dihydroxy-4-methylcoumarin, and naringenin. Meanwhile, quantitative monitoring of part of the components was illustrated in Figures 6 and 7. Simultaneously, the active ingredients by pure water extraction were as follows: oxymatrine, isoliquiritigenin, DL-stachydrine, cytisine, (+)-maackiain, 18-*β*-glycyrrhetinic acid, ginsenoside Rg3, 7,8-dihydroxy-4-methylcoumarin, and naringenin. Quantitative monitoring of part of the components was delineated in Figure 8.

## 4. Discussion

UCRCC is a malignant colonic disease and a multistep process with high mortality for which the accurate pathogenesis is inconclusive and well-appreciated effective therapy is limited. Recent advances have subscribed to the belief that continual inflammatory excitation structures a favourable background for UCRCC formation, providing proof that pivotal inflammatory mediators encompassing IL-6, TNF-*α*, NF-*κ*B, and IL-17 (also called IL-17A) coupled with Th17 cells are enriched in UC and colorectal cancer [8, 30–34]. Given its remarkable therapeutic capacity of CSD in UC [19, 20], the concept has fueled our hypothesis that CSD may mitigate the progression of UCRCC to a certain degree. Delightedly, in our study, CSD demonstrates an inhibitory effect on the release of these inflammation-related cytokines and secretion of Th17 cells coinciding with reduced occurrence of polyp/tumor and preferable well-being. Thereupon, the outcomes may help to develop a mind map for the investigation of mechanism and therapy with respect to UCRCC.

Apoptosis conducted in the intrinsic pathway, mainly by the mitochondrial apoptosis-induced channel, is an essential practice of programmed cell death characterized by cellular morphological changes and death [35, 36]. Bcl-2 is localized to the outer membrane of mitochondria, where it exerts a significant role in promoting cellular survival and opposing the actions of pro-apoptotic proteins such as mitochondria-cleaved caspase-3 and caspase-9. Mitophagy is the selective degradation of malfunctioning or damaged mitochondria via autophagy to retain the mitochondrial quality, thus making cells adapted to various types of stress. Accumulating evidence has delineated a fundamental role of mitochondrial energy production and apoptotic mechanism in the tumor initiation [12, 16, 37]. The lipid composition of mitochondrial membrane has been reckoned capable of regulating mitochondrial membrane permeability and thence, cell death [11, 12, 38]. Considering the multifaceted roles of mitochondria and intricate functions of mitophagy in tumorigenesis, care is exercised in the present study to decipher the role of the network comprising apoptosis, mitophagy, and inflammation responses in UCRCC and then to highlight innovative curative perception about UCRCC in support of the possibility that CSD can fine-tune the network.

Ultimately, our result that mitophagy and inflammation are positively joined to tumor progression in contrary to the fashion of apoptosis and CSD capsizes the trend remarkably may develop a new roadmap for the development of antitumor drugs for UCRCC.

## Data Availability

The data used to support the findings of this study are available from the corresponding author upon request.
